# High Lifetime Pregnancy and Low Contraceptive Usage Among Sex Workers Who Use Drugs- An Unmet Reproductive Health Need

**DOI:** 10.1186/1471-2393-11-61

**Published:** 2011-08-18

**Authors:** Putu Duff, Jean Shoveller, Ruth Zhang, Debbie Alexson, Julio SG Montaner, Kate Shannon

**Affiliations:** 1 British Columbia Centre for Excellence in HIV/AIDS, St. Paul's Hospital, 608-1081 Burrard Street, Vancouver, BC, V6Z 1Y6, CANADA; 2 School of Population and Public Health, University of British Columbia, 5804 Fairview Avenue, Vancouver, BC, V6T 1Z3, CANADA; 3 WISH Drop-In Centre Society, Vancouver, V6B 1S5, BC CANADA; 4 Department of Medicine, University of British Columbia, St. Paul's Hospital, 608-1081 Burrard Street, Vancouver, BC, V6Z 1Y6, CANADA

## Abstract

**Background:**

The objective of this study was to describe levels of pregnancy and contraceptive usage among a cohort of street-based female sex workers (FSWs) in Vancouver.

**Methods:**

The study sample was obtained from a community-based prospective cohort study (2006-2008) of 211 women in street-based sex work who use drugs, 176 of whom had reported at least one prior pregnancy. Descriptive statistics were used to estimate lifetime pregnancy prevalence, pregnancy outcomes (miscarriage, abortion, adoption, child apprehension, child custody), and contraceptive usage. In secondary analyses, associations between contraceptive usage, individual and interpersonal risk factors and high number of lifetime pregnancies (defined as greater than the sample mean of 4) were examined.

**Results:**

Among our sample, 84% reported a prior pregnancy, with a mean of 4 lifetime pregnancies (median = 3; IQR: 2-5). The median age of women reporting 5+ pregnancies was 38 years old [interquartile range (IQR): 25.0-39.0] compared to 34 years [IQR: 25.0-39.0] among women reporting 4 or fewer prior pregnancies. 45% were Caucasian and 47% were of Aboriginal ancestry. We observed high rates of previous abortion (median = 1;IQR:1-3), apprehension (median = 2; IQR:1-4) and adoption (median = 1; IQR:1-2) among FSWs who reported prior pregnancy. The use of hormonal and insertive contraceptives was limited. In bivariate analysis, tubal ligation (OR = 2.49; [95%CI = 1.14-5.45]), and permanent contraceptives (e.g., tubal ligation and hysterectomy) (OR = 2.76; [95%CI = 1.36-5.59]) were both significantly associated with having five or more pregnancies.

**Conclusion:**

These findings demonstrate high levels of unwanted pregnancy in the context of low utilization of effective contraceptives and suggest a need to improve the accessibility and utilization of reproductive health services, including family planning, which are appropriately targeted and tailored for FSWs in Vancouver.

## Background

To date, research on the sexual and reproductive health of female sex workers (FSWs) has primarily focused on vulnerability to sexually transmitted infections (STIs), particularly HIV infection [1]. However, FSWs also may be at high risk of pregnancy, as most are of reproductive age and many experience frequent vaginal intercourse without adequate access to contraceptives [2,3]. However, the broader reproductive health and pregnancy patterns of FSWs, particularly those who use drugs, have been largely neglected in research and public health interventions globally. Understanding the availability and use of contraceptives as well as pregnancy patterns among FSWs who use drugs is essential to develop more comprehensive, women-centred reproductive health services that promote positive maternal and child health and reduce adverse outcomes such as maternal mortality, unsafe abortions, the vertical transmission of HIV/AIDS, and poor birth outcomes for children of FSWs.

Access to reproductive health services, including contraceptives, prenatal care and mothering services [4], are a basic human right [5,6], and are particularly important for marginalized, drug-using women who may experience higher rates of maternal morbidity, mortality and negative obstetric, fetal and child outcomes as a result of drug use during pregnancy [7-10]. While research about access to reproductive health services among drug-using FSWs is scarce, a number of studies report that substance-using mothers experience limited or no access to prenatal care because of the stigma attached to maternal substance use during pregnancy [11-13]. Current child-centred policies in Canada have also been found to pose significant barriers to accessing appropriate reproductive health and treatment programs among women who use substances during pregnancy; many women avoid these services for fear of losing custody of their children [14]. The lack of recognition of the reproductive health needs and rights of women who use drugs limits their access to appropriate and non-judgmental reproductive health care services [15,16]. In addition to barriers posed by their drug use, FSWs' involvement in sex work may further limit their access to reproductive health services. Police surveillance and crackdowns have been found to displace street-based FSWs to peripheral areas, away from health care services [17,18]. Elsewhere, a lack of targeted services, expensive travel costs, health care service hours and sex work stigma have been identified as barriers to health and social services among FSWs [19], and could potentially restrict access to reproductive health services and impact levels of pregnancy and contraceptive usage among women in our sample.

Most previous studies on reproductive health practices and outcomes of FSWs have been among FSWs living in developing countries. Our literature search yielded no studies that explicitly focused on the reproductive health needs of street-based FSWs who use drugs in industrialized countries. Thus, we undertook a study of street-based FSWs living in Vancouver, Canada, to: (1) describe levels of contraceptive usage, prior pregnancy and pregnancy outcomes (including abortion, adoption, child apprehensions, miscarriage, child custody arrangements); (2) examine the correlates of having a high number of prior pregnancies (defined as having greater than the mean number of pregnancies (5+)).

## Methods

Data were obtained from a community-based prospective cohort of the BC Centre for Excellence in HIV/AIDS, in partnership with a local sex work agency, Women's Information Safe Haven (WISH) Drop-in Centre Society. The study approach has been described in detail previously [20]. Briefly, between 2006 and 2008, 252 street-based female sex workers were recruited using targeted outreach at solicitation spaces, based on mapping and time-location sampling. While this was not a random sample, time-location sampling has been increasingly used as a method for recruiting hard-to-reach populations at times and spaces where they congregate. The study's eligibility criteria included: self- identifying as a woman (including transgender), aged 14 years or older, actively engaged in street-level sex work and having used illicit drugs within the past month (excluding marijuana). Participants who consented and were enrolled in the study completed a baseline and 6-monthly follow-up interview-administered questionnaires by trained peer researchers (current or former street-based sex workers). Additionally, nurse-administered pre-test counseling and HIV screening using Biolytical Laboratories INSTI™ Rapid HIV Antibody Test (specificity 99.3%, sensitivity 99.6%) were conducted. At baseline, a detailed semi-structured questionnaire covering demographics, health service use, working conditions, violence, sexual and drug risk practices was administered. Participants received $25 remuneration for their time and expertise. Ethical approval for this research was received from the UBC/Providence Health Ethics Review Board.

### Statistical Analyses

Given our interest in lifetime pregnancy, our analyses were restricted to responses of women of reproductive age (18-49 years) who provided a valid response to "have you ever been pregnant". In total, 211 women met the above criteria and were included in the analyses. For our analyses of contraceptive usage, the entire sample of 211 women was used, as contraceptive usage is a relevant measure for all FSWs in our sample. For our analyses of pregnancy outcomes (such as adoption, custody, apprehensions and abortions), we restricted our sample to the 176 women who reported prior pregnancy. We excluded women who had never been pregnant as they were not as risk for these outcomes.

### Outcomes of interest

The following pregnancy outcomes were analyzed: prevalence of lifetime pregnancies; prevalence of abortion (a proxy of unwanted pregnancy); and miscarriage. Other pregnancy outcomes measured included adoption, child apprehension (defined as the removal of a child from his/her mother's custody by child protection services), has at least one child in her custody, and at least one child is in custody of family members. The analysis also examined the use of various types of contraceptives during the previous 6 months, including one or more of the following: barrier contraceptives only (male/female condom use); intrauterine device (IUD); hormonal contraceptives (birth control pill, Depo-Provera, Norplant); and, permanent contraceptives (tubal ligation, hysterectomy). We also accounted for FSWs' age (measured as a continuous variable), ethnicity, current drug use (any use of cocaine, crack cocaine, crystal methamphetamine), their exposure to 'social' factors (e.g., "having a partner who scores drugs for you"; having a regular partner that supports you financially). In bivariate analysis, associations between individual- and interpersonal- level variables and high number of lifetime pregnancies were examined. Higher number of lifetime pregnancies was defined as 5+ (operationalized as greater than the mean number of 4 pregnancies, versus less than the mean). Pearson chi-squared test was used to compare dichotomous, categorical variables and one-way analysis of variance (ANOVA) was used for comparison of continuous variables. P-values were generated using the Fisher's test of exact probability when one or more observations was less than or equal to five. We stratified pregnancy outcomes and contraceptive use by high (5+) versus low number of lifetime pregnancies. Variables with p values of < 0.1 were considered statistically significant and entered into a multivariable model. Variables were considered significant in multivariable analyses with an alpha cut-off of p < 0.05.

## Results

Of the 211 women eligible for this study, 176 (84%) women reported ever being pregnant. The median age of women reporting 5+ pregnancies was 38 years old [interquartile range (IQR): 25.0-39.0] compared to 34 years [IQR: 25.0-39.0] among women reporting 4 or fewer prior pregnancies. The median age of first sex work was 17 years of age [IQR:15-25]. Forty-five percent were Caucasian and 47% were of Aboriginal ancestry. Forty-seven (22%) reported being HIV positive, and 33(16%) were HIV positive, and taking Highly Active Antiretroviral Therapy (HAART). Approximately half (49%) of the women in our sample had non-commercial, intimate partners within the last 6 months of the interview.

Drug use was prevalent among our sample, with 103 (48.8%) having reported using heroin, 71 (33.7%) who used cocaine and 53 (25.1%) having reported intensive crack use (> 10 rocks per day) 6 months prior to the interview. The median age for initiation of injection drug use was 17.5 years [IQR = 15-23], and the median age of crack cocaine use was 20 years [IQR = 16-29.8].

### Pregnancy and child outcomes

The mean number of lifetime pregnancies for the entire sample (n = 211) was 4, with a median of 3 [IQR = 2-5]. Among FSWs who reported a prior pregnancy (n = 176), the median age of participants having 5 or more lifetime pregnancies was 38 years, compared to 34 years among women who reported having 4 or less pregnancies. The pregnancy outcomes for the 176 FSWs who reported ever being pregnant are reported in Table 1. Among FSWs who reported at least one prior pregnancy, forty-five percent reported a previous miscarriage (median = 1, IQR: 1-3) and 43% reported having a previous abortion (median = 1, IQR: 1-3). The proportion of women who reported having a prior miscarriage or induced abortion among the entire sample (n = 211) was 37% and 36% respectively. Thirty-two percent of women had one or more of their children apprehended by Child Welfare Services (median = 2, IQR: 1-4), while 20% currently had one or more of their children living with them (median = 2, IQR: 1-3). Twenty-eight percent of respondents said that they had one or more of their children being cared for by the child's father or another family member.

**Table 1:** Pregnancy outcomes among Female Sex workers (FSWs) who use drugs and reported at least one prior pregnancy

Pregnancy history	n = 176
	**Median, IQR**
Median number of pregnancies (entire sample, n = 211)	3 (2-5)
**Lifetime Pregnancy Outcomes among FSWs who reported prior pregnancy (n = 176)**	**n (%)** **Median, IQR**
Miscarriage	79 (45) 1 (1-3)
Abortion	76 (43) 1 (1-3)
Adoption	33 (19) 1 (1-2)
Child Apprehension	56 (32) 2 (1-4)
Currently supporting a child	35 (20) 2 (1-3)
Child in custody of family/father	49 (28) 2 (1-2)

The median age of initiation into sex work was higher among women who had five or more lifetime pregnancies (median = 18;[IQR = 14-22]) compared to women who had four or less lifetime pregnancies (median = 17; [IQR = 14-25]), though they were not significantly different (p = 0.92). Participants who had five or more prior pregnancies had a higher number (median = 7) of clients per week, compared to women who had four or less pregnancies (median = 5).

### Birth control practices

While consistent condom use by clients in our sample have previously been found to be high(72%) [21], only 14% of the 211 women included in our analysis reported relying on condoms alone as a method of birth control. There were no reports of female condom use in this sample. Nine percent reported having used injectable hormones (e.g., Depo-provera) and 1% reported that they had used the birth control pill. The median age of FSWs using any type of hormonal contraceptives (birth control pill, Depo-Provera, Norplant, injectable hormones) was 33 years [IQR:25-37]. Intrauterine devices (IUDs) were only reported by 1% of the women interviewed. Permanent contraceptives, including tubal ligation (16.6%) and hysterectomy (7.1%) were more common, though primarily among older FSWs (Median = 41 years; [IQR:36-45]) (Table 2). The median age among FSWs who did not use permanent contraceptives was 33.0 [IQR:25.0-38.0]. Older age(age was measured as a continuous variable), was significantly associated with permanent contraceptive use (p < 0.001).

**Table 2:** Contraceptive usage among 211 street-based Female Sex Workers (FSWs) who use drugs


**Barrier Contraceptive**	**n (%)**
Female condom	0 (0.0)
Condom only (for birth control)	30(14.2)
Condoms only with clients	139 (65.8)
**Female-controlled Contraceptives (excluding condoms)**	64 (30.3)
**Hormonal Contraceptive**	
Oral contraceptive pill	2 (1.0)
Intrauterine device (IUD)	3 (1.4)
Depo-provera/Injection	18 (8.5)
**Permanent contraceptive**	
Tubal ligation (sterilization)	35 (16.6)
Hysterectomy	15 (7.1)

In bivariate analysis, FSWs who reported tubal ligation had 2.5 greater odds of having a higher number of pregnancies (OR = 2.49; [95%CI = 1.14-5.45]). Similarly, FSWs who reported using permanent contraceptives (e.g., tubal ligation and hysterectomy) had 2.76 increased odds of having five or more pregnancies (OR = 2.76; [95%CI = 1.36-5.59]) compared to those who did not use permanent contraceptives. Alternatively, female-controlled contraceptives were not significantly associated with having five or more prior pregnancies (OR = 1.62; [95%CI = 0.86-3.03]). Individual-level drug risks and interpersonal risk factors for pregnancy (e.g., economic dependence on one's partner or having a partner who procures drugs) were not significantly associated with having five or more prior pregnancies (Table 3).

**Table 3:** Individual- and interpersonal- level factors associated with greater number of pregnancies (5+) among street-based Female Sex Workers (FSWs) who reported prior pregnancy (n = 176), with crude Odds Ratios, 95% Confidence intervals (95% CI) and p-values

	5+ pregnancies n (%)	Odds Ratio (95% CI)	*p - *value
***Demographic Characteristics***			
**Aboriginal**			
**yes**	42 (50.0)	1.49 (0.8-207)	0.19
**other**	42 (50.0)	(ref)	
**Caucasian**			
**yes**	35 (41.7)	0.75 (0.4-1.4)	0.33
**no**	49 (58.3)	(ref)	
***Contraceptive usage***			
Condoms only for birth control*			
**yes**	14 (17.9)	1.39 (0.6-3.2)	0.45
**no**	64 (82.1)	(ref)	
Female Controlled Contraceptives *			
**yes**	35 (44.3)	1.62 (0.9-3.0)	0.13
**no**		(ref)	
**Permanent Contraceptives** (Hysterectomy, Tubal ligation)*			
**yes**	30 (38.0)	2.76 (1.4-5.6)	< 0.01
**no**		(ref)	
Hysterectomy*			
**yes**	8 (10.1)	2.37 (0.7-8.2)	0.16
**no**		(ref)	
Tubal Ligation*			
**yes**	22 (28.2)	2.49 (1.1- 5.5)	0.02
**no**	56 (71.79)	(ref)	
Intrauterine Device (IUD)*			
**yes**	0.0 (0.0)	-	0.25
**no**		(ref)	
Hormonal Contraceptives*			
**yes**	6 (7.7)	0.44 (0.2-1.2)	0.10
**no**		(ref)	
Birth Control Pills*			
**yes**	0 (0.0)	-	0.50
**no**	78 (100.0)	(ref)	
Depo-Provera*			
**yes**	6 (7.7)	0.73 (0.3-2.2)	0.60
**no**		(ref)	
***Individual Level- Drug Use***			
**Cocaine†**			
**yes**	29 (34.5)	1.04 (0.6-1.9)	0.91
**no**		(ref)	
**Heroin†**			
**yes**	37 (44.0)	0.94 (0.5-1.7)	0.83
**no**		(ref)	
**Crystal Methamphetamine†**			
**yes**	6 (7.1)	0.57 (0.2-1.6)	0.28
**no**		(ref)	
**Crack cocaine†**			
**yes**	23 (27.4)	1.32 (0.6-2.2)	0.72
**no**		(ref)	
**Methadone†**			
**yes**	18 (21.4)	0.92 (0.5-1.9)	0.82
**no**		(ref)	
**Partner procures drugs**			
**yes**	23 (27.4)	1.13 (0.6-2.2)	0.72
**no**		(ref)	
**Economic dependence on partner**			
**yes**	23 (27.4)	0.96 (0.5-1.9)	0.90
**no**		(ref)	

* Currently Using  † Used within the last six month

## Discussion

Among this cohort of FSWs, we found a high prevalence of lifetime pregnancy, abortion and child apprehension, and low utilization of hormonal and insertive barrier contraceptives. On average, the women in this study reported four pregnancies during their lifetime, which is nearly three times the fertility rate of the general Canadian population [22].

Among our entire sample of 211 FSWs, 76 (36%) abortions were reported (median of 1, [IQR:1-3]). The self-reported prevalence of abortions among our cohort suggests a much higher level of unintended/unwanted pregnancy compared to the general Canadian population; the Canadian induced abortion rate is 14.1 per 1,000 women aged 15-44 [23]. Despite the lack of data on abortions among drug-using FSWs in resource rich countries, a few studies in low and middle-income countries have also reported high rates of abortion among FSWs [2,24]. For example, a study in Colombia found that 53% of FSWs interviewed reported having ever had an abortion [24]. Results from a Kenyan survey revealed an 86% prevalence of lifetime abortion among FSWs, with 50% of respondents reported having more than one [25]. Abortion data amongst the women enrolled our study are comparable to those in low-resource settings and suggest that despite legalized abortion and universal health care access in Canada, many women, particularly marginalized women, could benefit greatly from improved uptake of effective contraception and improved access to reproductive and sexual health care, including abortion services. Since our study does not capture abortions post-interview, our findings likely underestimate the true rate of lifetime abortions among FSWs. Additionally, though abortion rates can be used as a proxy for unwanted pregnancy, our findings likely underestimate the true rate, since access to abortion services may be limited among this population.

While evidence of female-controlled contraception among FSWs in our setting is scant, the limited use of contraceptives in our study is comparable to findings in resource-poor settings [1,2,26], and may suggest low access to female-controlled contraceptives and reproductive health services in this setting [27]. Other studies in our setting have found low utilization of health care services in general, due to the marginalization of FSWs and drug users, and their reluctance to use health and social services [27,28]. Avoidance of police and individual zoning restrictions (resulting from previous drug or solicitation charges) restrict FSWs' access to health services [29]. The high rate of child apprehension observed in our sample may further act as a barrier to seeking health care and social services [14]. Such policies that restrict access to health and social services can deny FSWs of enabling environments necessary to exercise their reproductive rights. Low access to reproductive health and mothering services (including antenatal care) may be of concern, considering the high rates of pregnancy among FSWs in our study. Contextual factors, such as poverty and homelessness, may reduce FSWs' ability to travel to clinics [19], or purchase hormonal contraceptives. Moreover, the instability arising from homelessness and illicit drug use may not be conducive to hormonal contraceptives such as the birth-control pill, which require routine and strict adherence, and annual or semi-annual prescription renewals. Homelessness has been associated with decreased access to health care services [30], and may also limit access to contraceptives. Illicit drug use may further exacerbate barriers to accessing contraceptives [24], although we found that illicit drug use and economic dependence on one's partner were not significantly associated with higher number of pregnancies. Additionally, perceptions of negative side effects (e.g., physical and emotional side effects; long-term health effects) of hormonal contraceptives may also contribute to their low uptake [31]. Additional studies are needed to elucidate the reasons for low use of hormonal and insertive contraceptives among FSWs.

The higher rate of condom use compared to hormonal contraceptives, particularly among younger FSWs, may reflect their knowledge of condoms' dual role in pregnancy and STI/HIV prevention. Low or no-cost condoms and their widespread availability also may contribute to the relatively higher use of condoms compared to other forms of contraceptives. Additionally, we found the rate of condom use (primarily by intimate/regular partners) to be much lower than the reported rate of consistent condom use by FSWs' clients from a previous study in our setting (72%) [21]. The low rates of condom use by intimate partners point to the need for dual protection from STIs/HIV and unwanted pregnancies. Long-lasting, female-controlled contraception methods, such as injectable hormones, may be effective in reducing unintended pregnancy. Permanent contraceptives usage was high among our sample, particularly tubal ligation and hysterectomy. The hysterectomy rate among our sample is exceptionally high when compared to the Canadian rate of 338 per 100,000 population [32]. The factors contributing to this prevalence in our study are unclear and warrant further attention.

This study has a number of limitations. The findings from this study may not be generalizable to FSWs working in other venues, such as bars, massage parlours and/or escort agencies. Given the sensitive nature of the topic, and our reliance on self-report data, the responses obtained in this study may be subject to social desirability bias. However, previous studies suggest that sex workers and drug users provide truthful accounts of their sex and drug use activities when questioned in a non-threatening environment [33]. Additionally, our study may underestimate the true rate of lifetime pregnancy and abortion among FSWs, as pregnancies post-interview are not captured. In the absence of data about pregnancy intention/desires, we used abortion rates as a proxy for unwanted pregnancy, which may limit our estimation of the true rate of unwanted pregnancy. However, induced abortion has been used as a proxy for unwanted pregnancy in other studies [34]. Finally, our small sample size (particularly our sample restricted to FSWs who had ever been pregnant) may have limited our statistical power to detect associations with high pregnancy levels.

## Conclusions

The high levels of unwanted pregnancy and underutilization of effective contraception suggests that FSWs who use drugs may not have access to knowledge or resources, such as contraceptives or reproductive health services, that allow them to have full control over their reproductive health. It is imperative to develop targeted services for FSWs that extend beyond HIV/STI prevention and comprehensively address reproductive health needs. The core principles of women-centred care, which emphasize women's autonomy, empowerment, safety, diversity and complexity as well the importance of social context in shaping reproductive health [35], need to be considered in order to better attend to the specific reproductive health needs of FSWs. Efforts to improve access and utilization of reproductive health services amongst this population also need to address other barriers, including the fear of child apprehension when help-seeking, particularly during pre- and post-natal periods. Comprehensive outreach services that include family planning and reproductive health services may hold promise for better serving this population [21,36].

## Competing interests

The authors declare that they have no competing interests.

## Authors' contributions

PD and KS designed the study. RZ conducted the statistical analysis. PD wrote the first draft of the manuscript and integrated suggestions from KS, JS and RZ. All authors made significant contributions to the conception and design of the analyses, interpretation of the data, and drafting of the manuscript, and all authors approved the final manuscript.

## Pre-publication history

The pre-publication history for this paper can be accessed here:

http://www.biomedcentral.com/1471-2393/11/61/prepub

## References

[B1] Delvaux T Crabbé F Seng S Laga M The need for family planning and safe abortion services among women sex workers seeking STI care in Cambodia Reprod Health Matters 2003 11 88 95 10.1016/S0968-8080(03)02163-3 12800706

[B2] Feldblum PJ Nasution MD Hoke TH Van Damme K Turner AN Gmach R Wong EL Behets F Pregnancy among sex workers participating in a condom intervention trial highlights the need for dual protection Contraception 2007 76 105 110 10.1016/j.contraception.2007.04.009 17656179

[B3] Chacham AS Diniz SG Maia MB Galati AF Mirim LA Sexual and Reproductive Health Needs of Sex Workers: Two Feminist Projects in Brazil Reproductive Health Matters 2007 15 108 118 10.1016/S0968-8080(07)29292-4 17512382

[B4] Gable L Gostin LO Hodge JG HIV/AIDS, reproductive and sexual health, and the law Am J Public Health 2008 98 1779 1786 10.2105/AJPH.2008.138669 18703431 PMC2636460

[B5] Cook RJ International human rights and women's reproductive health Stud Fam Plann 1993 24 73 86 8511808

[B6] UNFPA Supporting the Constellation of Reproductive Rights http://www.unfpa.org/rights/rights.htm

[B7] Wolfe EL Davis T Guydish J Delucchi KL Mortality risk associated with perinatal drug and alcohol use in California J Perinatol 2005 25 93 100 10.1038/sj.jp.7211214 15496968 PMC3349286

[B8] Broekhuizen FF Utrie J Van Mullem C Drug use or inadequate prenatal care? Adverse pregnancy outcome in an urban setting Am J Obstet Gynecol 1992 166 1747 1754 discussion 1754-1756 1615983 10.1016/0002-9378(92)91565-r

[B9] Minozzi S Amato L Vecchi S Davoli M Maintenance agonist treatments for opiate dependent pregnant women Cochrane Database Syst Rev 2008 CD006318 10.1002/14651858.CD006318.pub2 18425946

[B10] Shankaran S Lester BM Das A Bauer CR Bada HS Lagasse L Higgins R Impact of maternal substance use during pregnancy on childhood outcome Semin Fetal Neonatal Med 2007 12 143 150 10.1016/j.siny.2007.01.002 17317350 PMC3717561

[B11] Shankaran S Bauer CR Bada HS Lester B Wright LL Das A Health-care Utilization among Mothers and Infants Following Cocaine Exposure J Perinatol 0000 23 361 367 10.1038/sj.jp.7210946 12847529

[B12] Maupin R Lyman R Fatsis J Prystowiski E Nguyen A Wright C Kissinger P Miller J Characteristics of women who deliver with no prenatal care J Matern Fetal Neonatal Med 2004 16 45 50 10.1080/14767050412331283913 15370082

[B13] Pagnini DL Reichman NE Psychosocial factors and the timing of prenatal care among women in New Jersey's HealthStart program Fam Plann Perspect 2000 32 56 64 10.2307/2648213 10779236

[B14] Greaves L Varcoe C Poole N Morrow M Johnson J Pederson A Irwin L A motherhood issue: discourses on mothering under duress 2002 Ottawa: Status of Women Canada

[B15] Flavin J Paltrow LM Punishing Pregnant Drug-Using Women: Defying Law, Medicine, and Common Sense Journal of Addictive Diseases 2010 29 231 10.1080/10550881003684830 20407979

[B16] Roberts SCM Pies C Complex Calculations: How Drug Use During Pregnancy Becomes a Barrier to Prenatal Care Matern Child Health J 2010 10.1007/s10995-010-0594-7 PMC2904854 20232126

[B17] Shannon K Mapping violence and policing as an environmental-structural barrier to health service and syringe availability among substance-using women in street-level sex work Int J Drug Policy 2008 19 140 7 10.1016/j.drugpo.2007.11.024 18207725

[B18] Wood E Kerr T Small W Jones J Schechter MT Tyndall MW The impact of a police presence on access to needle exchange programs J Acquir Immune Defic Syndr 2003 34 116 8 10.1097/00126334-200309010-00019 14501805

[B19] Kurtz S Surrat H Kiley M Inciardi J Barriers to Health and Social Services for Street-Based Female Sex Workers Journal of Health Care for the Poor and Underserved 2005 16 2 345 61 10.1353/hpu.2005.0038 15937397

[B20] Shannon K Bright V Allinott S Alexson D Gibson K Tyndall MW Community-based HIV prevention research among substance-using women in survival sex work: The Maka Project Partnership Harm Reduct J 2007 4 20 10.1186/1477-7517-4-20 18067670 PMC2248179

[B21] Deering K Kerr T Tyndall MW Montaner JSG Gibson K Irons L Shannon K A peer-led mobile outreach program and increased utilization of detoxification and residential drug treatment among female sex workers who use drugs in a Canadian setting Drug and Alcohol Dependence 2001 113 46 54 10.1016/j.drugalcdep.2010.07.007 20727683

[B22] Statistics Canada The Daily, Monday, July 31, 2006. Births http://www.statcan.gc.ca/daily-quotidien/060731/dq060731b-eng.htm

[B23] Statistics Canada Induced Abortion Statistics http://dsp-psd.pwgsc.gc.ca/Collection/Statcan/82-223-X/82-223-XIE.html

[B24] Bautista CT Mejía A Leal L Ayala C Sanchez JL Montano SM Prevalence of lifetime abortion and methods of contraception among female sex workers in Bogota, Colombia Contraception 2008 77 209 213 10.1016/j.contraception.2007.11.013 18279693

[B25] Elmore-Meegan M Conroy RM Agala CB Sex Workers in Kenya, Numbers of Clients and Associated Risks: An Exploratory Survey Reproductive Health Matters 2004 12 50 57 10.1016/S0968-8080(04)23125-1 15242210

[B26] Todd CS Alibayeva G Sanchez JL Bautista CT Carr JK Earhart KC Utilization of contraception and abortion and its relationship to HIV infection among female sex workers in Tashkent, Uzbekistan Contraception 2006 74 318 323 10.1016/j.contraception.2006.04.006 16982233

[B27] Weber AE Tyndall MW Spittal PM Li K Coulter S O'Shaughnessy MV Schechter MT High pregnancy rates and reproductive health indicators among female injection-drug users in Vancouver, Canada Eur J Contracept Reprod Health Care 2003 8 52 58 12725675

[B28] Spittal PM Bruneau J Craib KJP Miller C Lamothe F Weber AE Li K Tyndall MW O'Shaughnessy MV Schechter MT Surviving the sex trade: a comparison of HIV risk behaviours among street-involved women in two Canadian cities who inject drugs AIDS Care 2003 15 187 195 10.1080/0954012031000068335 12856340

[B29] Shannon K Strathdee SA Shoveller J Rusch M Kerr T Tyndall MW Structural and Environmental Barriers to Condom Use Negotiation With Clients Among Female Sex Workers: Implications for HIV-Prevention Strategies and Policy American Journal of Public Health 2009 99 659 665 10.2105/AJPH.2007.129858 19197086 PMC2661482

[B30] Aidala AA Lee G Abramson DM Messeri P Siegler A Housing need, housing assistance, and connection to HIV medical care AIDS Behav 2007 11 101 115 10.1007/s10461-007-9276-x 17768674

[B31] Guendelman S Denny C Mauldon J Chetkovich C Perceptions of hormonal contraceptive safety and side effects among low-income Latina and non-Latina women Matern Child Health J 2000 4 233 239 10.1023/A:1026643621387 11272343

[B32] Canadian Institutes for Health Information, 2010 Health Indicators https://secure.cihi.ca/estore/productFamily.htm?pf=PFC1435&lang=en&media=0

[B33] Needle R Weatherby N Brown B Booth R Williams M Watters J Andersen M Chitwood D Fisher D Cesari H Braunstein M The reliability of self-reported HIV risk behaviors of injection and non-injection drug users Psychology Addictive Behaviour 1995 9 242 250

[B34] Kaye DK Mirembe FM Bantebya G Johansson A Ekstrom AM Domestic violence as a risk factor for unwanted pregnancy and induced abortion in Mulago Hospital, Kampala, Uganda Tropical Medicine & International Health 2006 1 90 101 16398760 10.1111/j.1365-3156.2005.01531.x

[B35] Midmer DK Does family-centered maternity care empower women? The development of the woman-centered childbirth model Fam Med 1992 24 216 221 1577215

[B36] Poole N Evaluation Report of the Sheway Project for High Risk Pregnant and Parenting Women 2000 Vancouver, British Columbia: British Columbia Centre of Excellence for Women's Health

